# Electroencephalography: Clinical Applications During the Perioperative Period

**DOI:** 10.3389/fmed.2020.00251

**Published:** 2020-06-09

**Authors:** Yi Sun, Changwei Wei, Victoria Cui, Meihong Xiu, Anshi Wu

**Affiliations:** ^1^Department of Anesthesiology, Beijing Chao-Yang Hospital, Capital Medical University, Beijing, China; ^2^Department of Anesthesiology, Washington University School of Medicine, St. Louis, MO, United States; ^3^Peking University HuiLongGuan Clinical Medical School, Beijing HuiLongGuan Hospital, Beijing, China

**Keywords:** EEG, depth of anesthesia, cognitive function, cerebral ischemia, prognostication

## Abstract

Electroencephalography (EEG) monitoring has become technically feasible in daily clinical anesthesia practice. EEG is a sensitive method for detecting neurophysiological changes in the brain and represents an important frontier in the monitoring and treatment of patients in the perioperative period. In this review, we briefly introduce the essential principles of EEG. We review EEG application during anesthesia practice in the operating room, including the use of processed EEG in depth of anesthesia assessment, raw EEG monitoring in recognizing brain states under different anesthetic agents, the use of EEG in the prevention of perioperative neurocognitive disorders and detection of cerebral ischemia. We then discuss EEG utilization in the intensive care units, including the use of EEG in sedative level titration and prognostication of clinical outcomes. Existing literature provides insight into both the advances and challenges of the clinical applications of EEG. Future study is clearly needed to elucidate the precise EEG features that can reliably optimize perioperative care for individual patients.

## Introduction

Electroencephalography (EEG) is a non-invasive, relatively inexpensive, and objective method for assessing neurophysiological function. There is increasing interest in the use of EEG monitoring in clinical practice, and its role in the surgical care pathway continues to evolve and expand. Although significant advancements have been made in perioperative care, adverse neurological outcomes remain an ongoing concern among clinicians. The overall picture of what happens to the brain during the perioperative period is unclear, particularly during procedures performed under general anesthesia.

EEG can provide important information about the cerebral cortex during the perioperative period, including detection of cerebral insults and depth of anesthesia. For example, predictable alterations to EEG can be identified in hypothermia, ischemia, and changes to depth of anesthesia (1). This paper will review current literature on the clinical applications of EEG in the perioperative period, including depth of anesthesia assessment and the prevention of perioperative neurocognitive disorders (PND). We also discuss current findings regarding the interpretation of raw EEG with administration of commonly used classes of anesthetic agents. Improved understanding of the advantages and limitations of EEG monitoring will benefit future surgical patients by promoting optimal, standardized perioperative care.

## Overview of EEG Analysis

**Figure 1:**
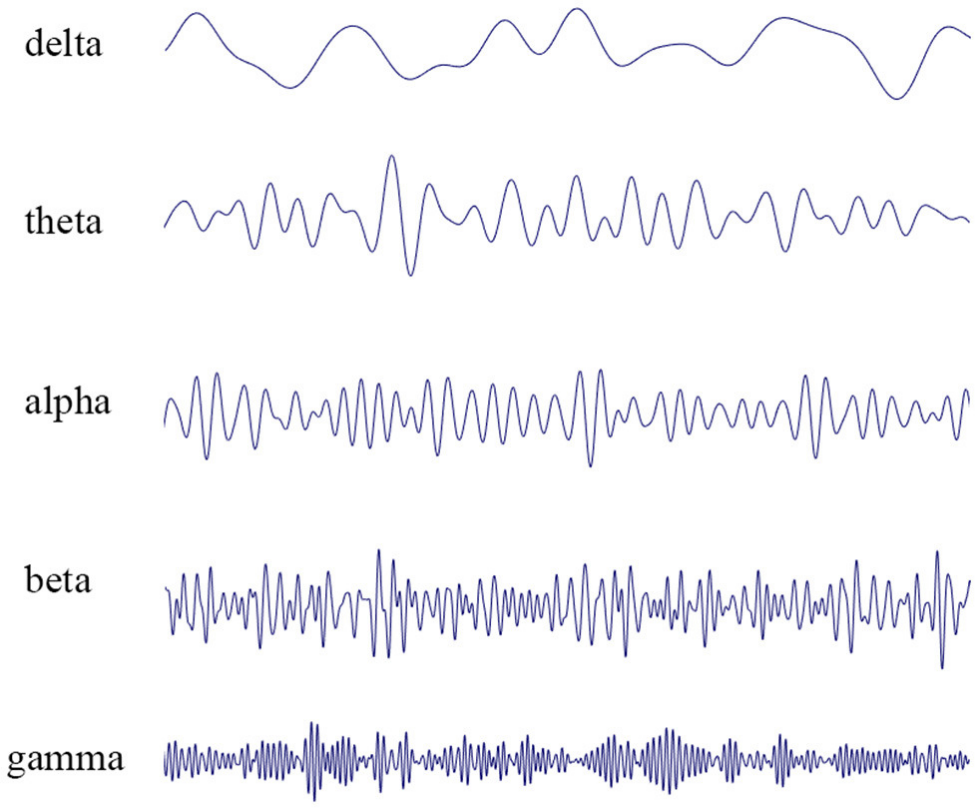
EEG recordings performed in patients. The waveform can be mathematically decomposed into different frequency bands: delta rhythm (0.5–3 Hz), theta rhythm (4–7 Hz), alpha rhythm (8–12 Hz), beta rhythm (13–30 Hz), and gamma rhythm (>30 Hz).

EEG represents the summation of excitatory post-synaptic potentials generated by individual neuronal cells in the cerebral cortex. In 1929, Hans Berger made the first observation of spontaneous electrical activity in the human brain (2). Berger defined two different frequency bands of wakefulness: alpha waves (8–12 Hz), predominant in the waking state with eyes closed, and beta waves (13–30 Hz), which often occur during mental concentration (Figure 1). In most of the population, closing the eyes results in a marked shift from predominately beta waves to alpha waves. These early findings were followed by the identification of theta waves (4–7 Hz) and delta waves (0.5–3 Hz), characteristic of sleep in adults, and gamma waves (>30 Hz), linked to cognitive function, information processing, and memory (3).

The first step in evaluation using EEG is the acquisition of EEG signal. Multiple electrodes, numbering 16–20 in the traditional “full” EEG, are distributed over the entire scalp (4). In many commercial processed EEG monitors designed for use in the perioperative setting, just 2–3 electrodes are placed across the patient's forehead. These electrodes effectively capture voltage fluctuations between different points on the scalp, transmitting through amplifiers and hard wire filters to yield the raw EEG signal (5).

EEG monitoring is very sensitive to artifacts and interference, and heavy disturbances can make gathering meaningful information especially difficult. Obtaining clean, raw EEG patterns should be the priority before performing data processing. In both clinical and research practice for example, care should be taken in proper skin preparation and conductive material is required in order to minimize impedance at the electrode-skin interface. Many factors are known to interfere with the EEG signals including ambient noise, power-line interference, EMG artifacts, ECG artifacts, eye-movement artifacts, and eye blinks (6). Researchers often combine the selection of a relatively “artifact-free” time segment with the use of various noise reduction filters and artifacts removal methods for improved reliability in EEG analysis.

Each EEG recording generates a vast amount of information for interpretation. The primary description of an EEG consists of amplitude, frequency, and wave shape. However, these basic criteria alone cannot comprehensively describe the multidimensional information present. To allow for more in-depth analysis, advanced time, and frequency parameters can be defined and extracted from raw EEG data.

Analysis in the time domain of EEG evaluates characteristics, changes, and pre-defined events of the waveform morphology over time. Average waveform length, waveform amplitude, slope sign change, and number of zero crossings are all examples of time domain EEG analysis (7). Of special interest in anesthesia is the occurrence of burst suppression, where a period of high- frequency sharp waveforms (burst) are followed by flat traces (suppression). Several accepted models have been developed to calculate such parameters including burst suppression ratio and time domain analysis, which have proven important in tracking transient waveform morphologies like epileptic spikes or sleep spindles (8).

The predominant frequencies of waveforms in the EEG are the focus of frequency domain analysis. A central technique in frequency domain analysis is the Fourier transform, a mathematical function used to decompose the raw EEG waveform into a sum of sine waves with discrete frequencies. The Fourier transform also outputs the relative contribution of each sine wave to the overall amplitude of the waveform. In this manner, power spectra with frequency on the x-axis and power, calculated as the square of the amplitude, on the y-axis can be plotted to visually represent the presence of each frequency bands in different brain states. Parameters including median frequency, peak frequency, and spectral edge frequency can then be calculated to further characterize the EEG (7).

Time-frequency analysis combines consideration of the time and frequency domains. Methods used in time-frequency analysis include short-time Fourier transform, wavelet transform, multitaper methods, and Hilbert-Huang transformation (9). Researchers may visualize these data with power spectra where the relative power at each frequency band, represented by color, is displayed as it changes with time.

## EEG in the Operating Room

Anesthesiologists often adjust anesthetic depth based on clinical signs, including blood pressure and heart rate. While changes in clinical signs can represent spinal neuronal responses to noxious stimulation, they are unable to directly reflect brain states or depth of anesthesia (10). Physiologic responses to anesthetics also varies in the population, and patients with overly light anesthesia levels have been found to lack corresponding hemodynamic changes (11). Identifying the appropriate depth of anesthesia for individual patients in an objective manner can prevent deleterious consequences of anesthetic under-dosing (e.g., intraoperative awareness) or anesthetic overdosing (e.g., perioperative neurocognitive disorders and long-term mortality). Gibbs et al. first reported the effect of anesthetic agents on EEG in 1937, forming the conceptual basis for the use of EEG to monitor the depth of anesthesia (DoA) (12). Since the 1990's, EEG has seen widespread clinical use for assessment of the depth of anesthesia and sedation (13).

### Processed EEG Monitoring for Depth of Anesthesia

Guidelines published by the Association of anesthetists of Great Britain and Ireland in 2016 recommend the use of depth of anesthetic monitors in patients undergoing total intravenous anesthesia with neuromuscular blocking to reduce intraoperative awareness (14). Most recently, the World Health Organization-World Federation of Societies of Anaesthesiologists (WHO-WFSA) International Standards for a Safe Practice of Anesthesia published in 2018 suggested the use of an electronic device (DoA) intended to measure brain function under general anesthesia, especially in patients at risk of awareness and postoperative delirium (15).

Several commercially available EEG-based monitoring devices are compared in Table 1. Generally, the raw EEG signal is captured via forehead electrodes and processed to derive a numerical index representing the anesthetic state. These index figures can then be used by the clinician to guide decisions.

**Table 1:** Commercial EEG-based monitoring systems for depth of anesthesia.

**Product**	**Utilized parameters**	**Scale**	**Clinical correlate**	**References**
Bispectral index (Covidien)	BSR; Relative beta activity; Synch-Fast-Slow	0–100	Awake: 100; Adequate anesthesia: 40–60; No brain activity: 0	(16–18)
AEP monitor/2 (Danmeter A/S)	ARX model; MLAEP latency and amplitude; BSR	AAI:0–100	Awake: 100; Adequate anesthesia: 15–20; Very deep hypnosis: 0	(19)
aepEX (Medical Device Management Ltd.)	MLAEP latency and amplitude	0–99	Awake: 100 Adequate anesthesia: 30–50; No brain activity: 0	(20)
Entropy Module (GE Healthcare)	Power spectrum Shannon function	RE:0–100 (nociception level) SE: 0–91 (depth of sedation)	Adequate anesthesia for both RE and SE parameters: 40–60 Need for additional analgesic: RE-SE difference ≥ 10	(21)
Narcotrend (MonitorTechnik)	Burst suppression; AR models; SEF, median frequency, spectral entropy, relative delta, theta, alpha, beta	Stages: A-F; Index: 0–100	Stages A (awake) to F (deep anesthesia), with 15 substages Index values: 100 (awake) to 0 (deep anesthesia), reflecting the continuous change of consciousness	(22)
PSA 4000 Monitor (Physiometrix, Inc)	Power spectrum;	PSI:0–100	Awake: 0 Adequate anesthesia:40–50 Eyes opening: 80	(23, 24)
Cerebral State Monitor (Danmeter A/S)	Alpha and beta ratio Alpha-beta difference Burst suppression	CSI:0–100	Awake: 90–100 Adequate anesthesia: 40–60 Deep: 10–40 Very deep: 0–10	(25)

*BSR, burst suppression ratio; MLAEP, middle latency auditory evoked potentials; RE, response entropy; SE, state entropy; ARX, autoregressive models with exogenous input; AAI, A-line autoregressive index; AR, autoregressive; SEF, spectral edge frequency; PSI, patient state index; CSI, cerebral state index*.

The first reported and the most widely-used depth of anesthesia monitor is the bispectral index, or BIS monitor (Covidien) (26). The BIS incorporates information from the time domain, frequency domain, and higher-order spectral subparameters. The subparameters extracted from the EEG recording are assigned weights in a proprietary multivariable model based on a clinical database of EEG changes correlated with behavioral assessment of hypnosis levels obtained from 1,500 healthy patients who received general anesthesia (13). The BIS monitor then outputs a single number on an index scale ranging from 0 (absence of brain activity) to 100 (awake). The BIS value correlates well with level of consciousness, with loss of consciousness generally observed to occur at BIS values between 68 and 75 (27). BIS values between 40 and 60 are considered indicative of adequate maintenance of general anesthesia for surgery (28). Since its development, further research has provided insight into the uses of the BIS. The multicenter B-Aware trial identified decreased incidence of intraoperative awareness with use of BIS-guided anesthesia (target BIS values, 40–60) in 2,463 high-risk surgical patients who received general anesthesia (16). A systemic review of 36 trials with a total of 7,761 high-risk surgical patients identified a lower risk of intraoperative awareness with use of BIS-guided anesthesia compared to clinical sign-guided anesthesia (29).

Several other EEG-based depth of anesthesia monitors have been developed and are approved for clinical use. The efficacy of these devices in clinical settings and populations continues to be evaluated, and each demonstrates advantages and limitations. While the aepEX monitor (Medical Device Management Ltd.) exhibited higher sensitivity and specificity compared to the BIS for detection of return of consciousness in pediatric patients, it was inferior to the BIS in distinguishing between levels of sedation in that population (17, 30). In a multicenter randomized trial, continuous monitoring patients with the PSA 4000 resulted in decreased use of propofol without increase in unwanted somatic events, hemodynamic instability, or intraoperative awareness compared to a standard practice group (18). In a study of 61 children aged 0–24 months, the Narcotrend device (MonitorTechnik) was found to exhibit an overall prediction probability of 0.8 in predicting sevoflurane concentration, with correlation in older children (31). In another study of children aged 12–17 years receiving propofol deep sedation, the Narcotrend Index helped to reduce recovery time, drug consumption, and episodes of undesired oversedation compared to clinical signs protocol (32).

Despite some studies demonstrating the benefit of using processed EEG monitoring during total intravenous anesthesia, no convincing evidence yet supports their application in anesthesia with volatile agents, when end-tidal anesthetic gas (ETAG) is also monitored. The B-Unaware trial did not find decreased incidence of intraoperative awareness with BIS-guided anesthesia compared to an anesthetic protocol based on ETAG in 2,000 patients considered high-risk for intraoperative awareness (33). The subsequent multi-center BAG-RECALL trial, conducted in a similar high-risk group of 6,041 patients, found a lower incidence of intraoperative awareness in ETAG-guided patients than BIS-guided patients (34).

Knowledge and use of current EEG-based monitors in the clinical setting has several limitations. Surgical populations and anesthesia protocols, with and without EEG guidance, vary significantly between completed studies. The predictive probabilities of these tools are also generally less reliable in pediatric patients and especially infants, as reference EEG databases have been derived from adult populations (35). Inappropriately low index values are often reported by depth-of-anesthesia monitors in pathological states including cerebral hypoperfusion (36), hypoglycemia (37), and hypothermia (38), conditions which are important to detect perioperatively. Lastly, index values may increase with administration of ketamine (23) and nitrous oxide (39), underestimating the true depth of anesthesia.

### Raw EEG for Depth of Anesthesia

While the information from a single processed EEG index may be limited, researchers have begun to explore the use of raw EEG to identify and evaluate brain states under anesthesia. In 2019, the American Society for Enhanced Recovery and Perioperative Quality Initiative Joint Consensus Statement recommend anesthesiologists to interpret basic EEG, including raw waveform and spectrogram (40). Anesthetic agents are believed to act at specialized receptors in specific brain circuits, resulting in changes to EEG features. With ongoing advances in neuropharmacology and neurophysiology, it may become possible to recognize drug-specific patterns in raw EEG using high-density EEG recording. This approach may allow identification of shared and distinct mechanisms of anesthetic agents (8). In this section, we review current knowledge of neuropharmacology and clinical electrophysiology of several widely-used anesthetic agents.

### Propofol

Propofol, a GABA_A_ agonist, is the most commonly used anesthetic for intravenous general anesthesia. It is also frequently administrated for sedation in the intensive care unit. Propofol administration has been associated with distinct changes to EEG, including increased beta oscillation during paradoxical excitation, which occurs at an early stage of propofol induction (19). With increase in the propofol infusion rate, the EEG shifts into the alpha range, with strengthening of synchronous alpha activity in the frontal lobes at deeper level of propofol anesthesia (20). This frontal alpha signature represents synchronous alpha-oscillation activity in the cortex and thalamus. Global coherence analysis concurs with these findings, demonstrating a transition of coordinated alpha activity from the occipital lobes of awake subjects to the frontal lobes following propofol administration and loss of consciousness (21). During this transition, low frequency oscillations (<1 Hz) have been identified in the local field potential, suggesting the potential role of propofol in disrupting the spatial and temporal organization of network dynamics (22).

The changing relationship between alpha amplitudes and low frequency oscillations may also reflect differences in states of consciousness. During the transition from consciousness into unconsciousness, alpha amplitudes are maximal at the troughs of low frequency oscillations. In profound unconsciousness, alpha amplitudes become maximal at the peaks of low frequency oscillations (24).

Deep anesthesia induces emergence of burst suppression, while complete suppression of EEG is associated with excessive depth of anesthesia (25). Burst suppression appears with propofol-induced unconsiousness and its onset does not interfere with the dominant alpha rhythm (41). Burst suppression has been shown to exhibit substantial asynchrony across different cortical regions. In some cases of lighter anesthesia, the pattern may be restricted to certain regions while others exhibit continuous activity (42).

With emergence from propofol anesthesia, changes in EEG patterns are generally reversed. Recovery of consciousness is marked by a decrease in coherent frontal alpha oscillations and low-frequency power, accompanied by reappearance of coherent occipital alpha oscillations (24). Maximal alpha amplitudes are again observed at the troughs of low-frequency oscillations during this transition between unconsciousness and consciousness (24). A transient brain state with large, spatially distributed, slow sensory-evoked potentials has also been observed prior to recovery of behavioral responsiveness following emergence from propofol anesthesia (43).

### Volatile Anesthetics

Volatile anesthetic agents are used widely for the induction and maintenance of anesthesia, especially in pediatric patients. Isoflurane and sevoflurane induce similar sequences of EEG changes as general anesthesia is deepened. During light isoflurane anesthesia, EEG waveforms are shallow and fast. With increased depth of anesthesia, the EEG waveforms slow and deepen in amplitude, and burst suppression may begin to appear. The flat traces during burst suppression become markedly more pronounced as isoflurane is increased (25). Similar to propofol, unconsciousness induced by sevoflurane is associated with coherent alpha rhythms in the frontal lobe and slow oscillations suggestive of synchronization between the thalamus and the cortex. Sevoflurane administration is also associated with increased power and coherence in the theta range (44).

EEG responses to volatile anesthetic exhibits age-dependent variability. Infants given sevoflurane anesthesia have been observed to exhibit slow oscillations but lack the predominant and coherent alpha activity in the frontal lobes seen in adults (45). At around 4–6 months of age, theta and alpha range oscillations emerge under maintenance of anesthesia (45). These waveforms diminish with reduction of end-tidal sevoflurane and emergence from anesthesia (45). From 4 to 10 months of age, alpha oscillation power increases in the frontal lobes (46). Alpha rhythm coherence, characteristic of an inactive brain under sevoflurane anesthesia in adults, begins to develop in children around 10–12 months of age (46, 47). These age-related EEG changes likely reflect processes including synaptogenesis, differences in glucose metabolism, and progressive myelination of the cortex in the developing btain (45).

In both children (48) and adults (49), epileptiform EEG activity has been reported with high-dose sevoflurane induction. Controlled hyperventilation in adult patients receiving rapid sevoflurane induction appears to increase incidence of these epileptiform discharges (49). This effect was reduced but not eliminated in children receiving shortened exposure (<5 min) to 8% sevoflurane at anesthesia induction (50).

### EEG and Nitrous Oxide

Nitrous oxide is a sedative hypnotic that potently inhibits the N-methyl-D-aspartate (NMDA) receptor. Unlike the EEG response to propofol and volatile anesthetics, administration of nitrous oxide is associated with decreased alpha and delta activity in the frontal lobes and increased high beta oscillations (51, 52).

While nitrous oxide alone is not considered sufficient to produce general anesthesia, it is commonly combined as an adjunct with volatile anesthetic, oxygen, and air in clinical practice. The observed clinical effect of nitrous oxide on EEG is thus altered by concurrently administered agents. In a small study of 15 surgical patients, addition of nitrous oxide during steady-state halothane anesthesia resulted in a progression from delta waves to theta waves, then spindle-type waves similar in conformation to the original waveforms induced by halothane (53). In that study, a second admixture of nitrous oxide given 20–30 min later resulted in variable EEG responses suggestive of acute drug tolerance. These changes included an abbreviated progression of theta waves followed by spindles, continuous delta waves, and spindle-type EEG waves only (53). In a separate retrospective study, patients consistently exhibited large amplitude, slow delta oscillations during the transition from maintenance sevoflurane anesthesia to facilitated emergence with over 60% nitrous oxide and total flow rate of >4 L/min (54).

### EEG and Ketamine

Ketamine, an NMDA antagonist, is used commonly in clinical anesthesia. At subanesthetic doses, ketamine has been observed to induce gradual dissipation of alpha power, with significant decreases in the precuneus and temporal-parietal junction (55, 56). A gamma-burst EEG pattern, consisting of alternating slow delta and gamma waves, has also been frequently observed with ketamine administration. This pattern is commonly followed by increased theta and decreased alpha/beta oscillations (57). At anesthetic doses of ketamine, theta power increases in the frontal lobes and reductions in anterior-to-posterior alpha connectivity appears to occur in a dose-dependent manner (55).

### EEG and Dexmedetomidine

Dexmedetomidine is used most often for light-to-moderate sedation in intensive care settings. Unlike sedation achieved with other anesthetic agents, patients can be easily aroused from dexmedetomidine-induced unconsciousness. Dexmedetomidine likely acts as a selective agonist at alpha-2 adrenergic receptors, altering arousal status via inhibition of the ascending arousal system (58). On EEG, dexmedetomidine administration has been observed to elicit spindle oscillations with maximum power and coherence of ~13 Hz in the beta range (59, 60). These spindle patterns exhibit similar spindle density, amplitude, and frequency compared to sleep spindles, but are longer in duration. These findings have clinical correlation with the observation that dexmedetomidine induces a state similar to non-rapid eye movement sleep (60).

### EEG and Prevention of Perioperative Neurocognitive Disorders

Surgery and anesthesia can significantly impact cognitive function and performance, especially in elderly patients. PNDs are diagnosed in the preoperative or postoperative period and as defined include baseline cognitive impairment, acute events such as development of delirium, and the development of cognitive dysfunction up to 30 days and 12 months after surgery (61). Patients with preexisting cognitive dysfunction are at greater risk for developing a PND. Postoperative cognitive decline is closely associated with increased mortality and other adverse outcomes (62). Persisting symptoms decrease quality of life, and patients experiencing cognitive decline postoperatively are at higher risk for later developing Alzheimer's disease (63).

EEG has important clinical application in PND prevention. The European Society of Anesthesiology currently recommends EEG-guided anesthesia monitoring for the prevention of postoperative delirium and cognitive decline (64). In a trial of 921 elderly adults undergoing general anesthesia for non-cardiac surgery, patients were randomized to receive BIS-guided anesthesia in which anesthesia was adjusted to maintain the BIS value within a recommended range of 40–60 or standard anesthesia care. Patients who received BIS-guided anesthesia had lower rates of postoperative delirium and cognitive decline at 3 months compared to routine care (65). In a trial of 1277 patients, patients who received anesthesia with intraoperative BIS monitoring had lower delirium incidence but no difference in the rate of cognitive dysfunction at 90 days. BIS-guided patients experienced fewer episodes of BIS values <20 during general anesthesia, suggesting deep anesthesia as a possible precipitant of postoperative delirium (66). Longer durations of intraoperative EEG suppression is associated with postoperative delirium (67), and patients with EEG suppression at lower concentrations of volatile anesthetic are more likely to develop postoperative delirium (68). A meta-analysis incorporating these studies found that processed EEG monitoring reduced the incidence of postoperative delirium in patients over 60 years of age (69). However, the recently completed ENGAGES trial found no difference in postoperative delirium incidence within 5 postoperative days in patients receiving EEG-guided anesthesia and those receiving standard anesthesia care (70). An updated meta-analysis including ENGAGES trial revealed no statistically significant reduction of EEG-based monitor on postoperative delirium risk (71). These findings no doubt elicited a huge discussion. Experts believed that EEG-guided group in ENGAGES trial failed to receive appropriate anesthesia, since that difference of anesthetic consumption between groups was small (40) and the duration of burst suppression in EEG-guided group exceeded the threshold relating to delirium occurrence (72). Further investigation is needed to clarify the role and limitations of EEG in PND prevention.

While not yet used in current clinical practice, EEG has emerging potential for detection of cognitive dysfunction. In prospective studies, postoperative delirium is associated with higher delta power in the frontal lobes during eyes-closed EEG recording (73, 74). Functional connectivity between different regions of the cortex is also disrupted in delirium. In cardiac surgery patients with delirium, alpha band functional connectivity decreases while delta band connectivity increases in the frontal regions (75, 76). Older adults with preoperative cognitive dysfunction exhibit have been shown to less intraoperative frontal alpha power during general anesthesia than their peers, suggesting a role for EEG monitoring in identifying patients at increased risk of developing PNDs (77).

### EEG and Detection of Cerebral Ischemia During Surgical Procedures

Patients face significant risk for cerebral ischemia during and after major surgery, especially when undergoing cardiac procedures. Many patients in the aging surgical population may be predisposed to experiencing cerebral ischemia due to pre-existing cerebral vascular disease. In these patients, a higher mean arterial blood pressure (MAP) is required to perfuse narrowed arteries and arterioles in the brain (78). Other common causes of perioperative cerebral ischemia include hypoperfusion during cross-clamping, impaired cerebral autoregulation, embolism, hypoxia, and anemia (79, 80).

Intraoperative EEG may be used to aid detection of cerebral ischemia, but its role is not yet well-defined (78). EEG primarily detects activity of the cortex, with limited-to-no efficacy in probing deeper brain regions which can also experience ischemia. One of the most commonly reported EEG features of cerebral ischemia is the attenuation of alpha and beta wave amplitudes and the enhancement of theta and delta wave amplitudes (81). Use of EEG for detection of cerebral ischemia has been an area of interest in carotid surgery (82). The clamping of the carotid arteries during CEA procedures and subsequent vascular shunting may cause insufficient blood flow to the ispsilateral cerebral hemisphere and cause intraoperative stroke. Carotid artery clamping resulted in around 20% decreases in the BIS both in the ipsilateral and contralateral sides (83). Burst suppression and isoelectric patterns are also indicators of cerebral ischemia. In patients undergoing aortic hemiarch replacement, abrupt loss of electrocerebral activity was recorded immediately after circulatory arrest (84). In this light, for certain surgical procedures that cause dramatic fluctuation in cerebral blood flow, changes of EEG provide alarms to clinicians. However, since anesthetic administration produces similar EEG patterns, clinicians should pay more attention to the exclusive use of EEG on detecting ischemic insults (80). While EEG is considered to have potential for lowering intraoperative stroke risk, it is not currently recommended for routinely use (85).

## EEG in the Intensive Care Unit

### EEG and Depth of Sedation

Despite monitoring depth of anesthesia during surgery, EEG is expanded to determine sedation levels in critically ill patients. The PADIS Guidelines in 2018 suggested to maintaining light sedation states in critically ill, mechanically ventilated adults (conditional recommendation, low quality of evidence). And they ungraded stated that “Sedation that is monitored with BIS compared with subjective scales may improve sedative titration when a sedative scale cannot be used” (86).

### EEG and Clinical Outcomes Prognostication

In cases of significant permanent or irreversible damage to the brain, continuous EEG (cEEG) monitoring may hold significant potential for outcomes prognostication. Post-anoxic encephalopathy after cardiac arrest is a condition in which cEEG can be useful in predicting neurological outcomes (87). In a retrospective study of cEEG in the first 12–72 h after cardiac arrest, continuous or nearly-continuous patterns at 12 h were associated with recovery of consciousness. Isoelectric patterns (voltage <2 μV) at 24 h and EEG suppression (voltage 2–10 μV) at 48 h after stroke were associated with failure to recover consciousness (87).

There is emerging evidence supporting burst suppression as a marker for hypoxic-ischemic brain damage. In post-anoxic encephalopathy after cardiac arrest, burst-suppression at both 24 and 48 h after stroke was associated with failure to recover consciousness after the initial insult (87). Burst suppression with a pattern of identical bursts was found to be associated with the most widespread patterns of damage on postmortem histopathology in a series of 11 patients who died following anoxic coma (88).

While gaining attention in research, cEEG is not easily interpretable by non-neurologists. Methods for processing raw EEG data such as amplitude-integrated EEG (aEEG) may aid in clinical adoption for outcomes prognostication. The lack of normal trace development within 36 h, status epilepticus, and burst suppression recorded by aEEG were considered indicative of “invariably poor” prognoses (89). In a retrospective study of 61 patients who survived out-of-hospital cardiac arrest but remained comatose following resuscitation, aEEG was used effectively in predicting neurological outcomes (90). In that study, patients were categorized as C1 if they regained continuous normal voltage on aEEG within 12 h following return of spontaneous circulation (ROSC). Of 20 C1 patients, 95% experienced a good neurological outcome of a cerebral performance category score between 1 and 2. Patients who were categorized as C4, indicating they experienced burst suppression at any time post-ROSC, universally experienced poor outcomes (90). In a retrospective study of 63 out-of-hospital cardiac arrest patients, epileptiform activity, or cerebral inactivity detected on BIS at any time post-ROSC was also found to be predictive of poor outcomes (91).

## Challenges and Perspectives

There are still many obstacles and challenges to the widespread, effective adoption of EEG monitoring in the perioperative period. First, the generalizability of existing evidence is not clear as age-related changes profoundly affect the brain and its response to physiological insults. Second, the complexity of raw EEG signal challenges the feasibility of raw EEG monitoring and interpretation in the clinical anesthesia setting. Researchers currently employ multiple disparate algorithms and analysis methodologies to investigate perioperative EEG patterns. There is no unified standard for the analysis of EEG features and its practical applications perioperatively, and any such application would likely require extensive training of medical personnel.

## Conclusion

In this review, we survey the clinical applications of EEG in the operative room and intensive care units, including assessment of depth of anesthesia, prevention of perioperative neurocognitive disorders, detection of cerebral ischemia, assessment of sedative states, and clinical outcome prognostication. We also discuss current knowledge of the effect of major anesthetic agent classes on EEG. Although with limitations, EEG has great potential as an objective, non-invasive tool in clinical and research settings. As the understanding of its effective use in the perioperative period improves, we believe that EEG can help provide customized delivery and improve outcomes of perioperative management for patients.

## Author Contributions

YS, CW, and VC drafted the manuscripts. CW, VC, MX, and AW contributed to critical revision. All authors reviewed the manuscript and approved the final version.

## Conflict of Interest

The authors declare that the research was conducted in the absence of any commercial or financial relationships that could be construed as a potential conflict of interest.
